# Bacterium of one thousand and one variants: genetic diversity of *Neisseria gonorrhoeae* pathogenicity.

**DOI:** 10.1099/mgen.0.001040

**Published:** 2023-06-07

**Authors:** Kacper Kurzyp, Odile B. Harrison

**Affiliations:** ^1^​ Sir William Dunn School of Pathology, University of Oxford, Oxford, UK; ^2^​ Department of Biology, University of Oxford, Oxford, UK; ^3^​ Nuffield Department of Population Health, University of Oxford, Oxford, UK

**Keywords:** genome, immune evasion, *Neisseria gonorrhoeae*, pathogenesis, variation

## Abstract

The bacterium *Neisseria gonorrhoeae* causes the sexually transmitted infection gonorrhoea. Although diverse clinical manifestations are associated with gonorrhoea, ranging from asymptomatic through to localized and disseminated infection, very little is known about the bacterial determinants implicated in causing such different clinical symptoms. In particular, virulence factors, although defined and investigated in particular strains, often lack comprehensive analysis of their genetic diversity and how this relates to particular disease states. This review examines the clinical manifestations of gonorrhoea and discusses them in relation to disease severity and association with expression of particular virulence factors including PorB, lipooligosaccharide (LOS) and Opa, both in terms of their mechanisms of action and inter- and intra-strain variation. Particular attention is paid to phase variation as a key mechanism of genetic variation in the gonococcus and the impact of this during infection. We describe how whole-genome-sequence-based approaches that focus on virulence factors can be employed for vaccine development and discuss whether whole-genome-sequence data can be used to predict the severity of gonococcal infection.

## Data Summary

The *Neisseria gonorrhoeae* FA1090 genome was downloaded from the PubMLST database (https://pubmlst.org/). The genome was annotated using Proksee (https://proksee.ca/). Analysis of the source of gonococcal genomes was performed using the PubMLST database (https://pubmlst.org/neisseria).

**Impact Statement**

In order to combat *Neisseria gonorrhoeae *, we need to fully understand the bacterium causing this disease, in particular how it continually eludes the immune response and is so successful at causing infection. However, despite decades of research, there is still much to learn about this versatile pathogen. Here, we provide a comprehensive review of what is known about *N. gonorrhoeae* disease manifestations, pathogenesis and the virulence determinants associated with this phenotype, with a particular focus on genetic variation.

## Introduction

*Neisseria gonorrhoeae* (the gonococcus) is the causative agent of the sexually transmitted infection (STI) gonorrhoea, which affects over 86.9 million people every year [1]. The bacterium causes acute inflammation of the genitourinary tract, characterized by recruitment of phagocytic cells to the site of infection [2]. Lower genitourinary tract infection is, however, not the only site of gonococcal tropism, as the gonococcus is known to also infect the rectum, throat, eyes, upper genitourinary tract and, in rare cases, the bloodstream, which may lead to establishment of distant skin and joint colonization [3–6].

Painful inflammation of the genitourinary tract is only one consequence of infection. In some cases, gonococci ascend to infect the upper genital tract, potentially leading to infertility if untreated. This is due to scarring associated with chronic neutrophilic inflammation with untreated infection in women. Resultant pelvic inflammatory disease (PID) is associated with significant pain, and increased risk of miscarriage and extrauterine pregnancy [1, 7, 8]. In both sexes, infection can spread, leading to septicaemia, skin lesions and arthritis in a condition known as disseminated gonococcal infection (DGI) [9, 10]. In addition, gonococcal infection has been associated with an increased risk of contracting human immunodeficiency virus (HIV) – which may be due to recruitment of lymphocytes to the urogenital tract [6, 11].

Improved understanding of *N. gonorrhoeae* virulence and its determinants enhances our ability to identify alternative treatment strategies – potentially resulting in gonococcal-specific antimicrobials [12–14]. Furthermore, identification of new virulence factors and understanding their variation will facilitate the discovery of conserved immunogenic structures – expanding the list of potential vaccine targets [15–18]. Here, we review current understanding of gonococcal host–pathogen interactions. First, we consider the different disease phenotypes exhibited by the gonococcus and how gonococcal infection severity is classified. We then examine what is known about individual virulence factors, their functions and their intra- and inter-strain genomic variability. Finally, we describe recent advances in the methodology for identification of gonococcal virulence factor diversity, and the challenges associated with bioinformatic analyses of whole-genome-sequence data. To cause disease, a pathogen has to express determinants that permit niche establishment and immune evasion, ultimately causing damage to the host as it tries to eliminate the pathogen and maintain homeostasis. Understanding these processes is essential to developing new ways to treat gonococcal infections, especially in the light of growing antimicrobial resistance.

## Gonococcal pathology is difficult to define and correlate with bacterial genetics

### *N. gonorrhoeae* has several clinical manifestations that cannot be easily categorized in terms of severity

Gonorrhoea is characterized by infection of the lower genitourinary tract mucosa leading to urethritis in males and cervicitis in females [1, 19, 20]. Pathology includes inflammation with a neutrophilic purulent exudate [21]. This is the classical manifestation of gonorrhoea, but the severity of pathology should be robustly characterized to allow comparisons to be made on the bacterial genetic determinants implicated in each clinical scenario in terms of human pathogenesis.

Asymptomatic infection is typically recognized as the ability to recover viable gonococci from the lower reproductive tract of a patient without symptoms of pain or visible inflammatory exudate [8, 22]. It can be detected during routine STI screening. It is estimated that 50 % of gonococcal infections in women are asymptomatic compared to a much lower proportion in men (though this gender disparity has been questioned) [8, 22]. The term asymptomatic infection does not differentiate colonization from subclinical inflammation, making the issue of investigating pathogenesis challenging. Thus, asymptomatic infection has a vague pathological definition, making severity classification difficult.

In addition, there is extensive evidence indicating that asymptomatic infection is not without pathology. Firstly, the interaction of the gonococcus with other STI-causing organisms is of growing concern. *N. gonorrhoeae* infection is known to cause inflammation, epithelial damage and recruitment of Th17 polarized CD4^+^ T cells to the urogenital mucosa and, as a result, provides opportunities for HIV acquisition [23–25]. Furthermore, clinical data suggest frequent co-infection with *Chlamydia trachomatis *; in a murine model of infection, co-infection was associated with a higher gonococcal burden and increased neutrophil recruitment that may translate to more severe symptoms [26, 27]. Therefore, asymptomatic infections may have an important clinical role in STI epidemiology.

Secondly, both symptomatic and asymptomatic infection can ascend if left untreated [7, 8, 28, 29]. The most common sequela of untreated gonorrhoea is PID, which is defined as bacterial infection associated with inflammation of the upper female genital tract [8]. This includes endometritis, salpingitis and oophoritis, and is most commonly associated with significant lower abdominal pain that intensifies on menstruation with serious sequelae (Table 1) [30]. Some suggest that the mechanism of ascending infection may be purely mechanical, through retrograde menstruation or sexual intercourse [8, 31]. However, attachment is vital for bacterial colonization. Differential expression of carcinoembryonic antigen-related adhesion molecule (CEACAM)1 and CEACAM5 has been shown in human female reproductive tract samples, with CEACAM1 expressed in the uterus and endocervix (upper genital tract) and CEACAM5 expressed in the ectocervix (lower genital tract) [32]. Reciprocally, Opa_CEA_ expressing gonococci have been shown to colonize the murine uterine horn, equivalent to the human uterine body, in human CEACAM1 transgenic mice [32], indicating that expression of particular Opa alleles, perhaps by a subpopulation of gonococci, may be associated with PID development. However, data on association of gonococcal genetic variants with PID are limited, likely due to difficulties in obtaining samples from the upper genital tract. However, the recent establishment of a murine female genital tract infection model transfected with human CEACAM receptors may permit more detailed studies into PID pathology [32].

**Table 1.** Summary of gonococcal disease manifestations and their severity −, No long-term sequelae; *, minor long-term sequelae; **, significant long-term sequelae; ***, serious long-term sequelae.

Clinical manifestation	Gender affected	Mechanism	Symptoms	Long-term sequelae	Reference
Lower genital tract symptomatic infection	Both (males higher proportion)	Sexual transmission	Painful inflammation of genitourinary tract; urethral stricture and pain on urination in males; increased risk of acquisition of HIV; may ascend/spread (PID, DGI)	*/**	[1, 19–21]
Lower genital tract asymptomatic infection	Both (females higher proportion)	Sexual transmission	Potentially none, but can become symptomatic or ascend/spread (PID, DGI)	−	[8, 22, 24, 25]
Pharyngitis	Both	Sexual transmission	Inflammation of pharynx (often asymptomatic)	*	[5, 39]
Pelvic inflammatory disease (PID)	Female	Ascension of lower genital tract infection	Severe abdominal pain; scarring of the reproductive tract; increased risk of extrauterine pregnancy, stillbirth and spontaneous abortion; infertility	**/***	[7, 8, 29, 30]
Disseminated gonococcal infection (DGI)	Both	Systemic infection spread from other sites	Septic arthritis and joint pain; septic shock in rare cases	**/***	[3, 9, 10]
Ophthalmia neonatorum	Both (neonates)	Eye infection in the newborn from infected mother	Corneal scarring, globe perforation and permanent blindness	***	[40]
Gonococcal conjunctivitis	Both	Eye infection in adults from infected urine or genital secretions	Corneal scarring, globe perforation and permanent blindness	***	[41]

The most invasive manifestation of *N. gonorrhoeae* infection is DGI. Here, the bacterium disseminates to the bloodstream. Exposure to bacterial pro-inflammatory molecules can trigger life-threatening sepsis in rare cases; more common is occurrence of skin lesions or septic arthritis, which unlike other joint inflammatory conditions are non-symmetrical and difficult to predict based on patient history [3, 9, 10]. The adaptations necessary for systemic spread are serum and complement resistance; this was observed in isolates recovered from patients with DGI [33, 34]. The outer membrane PorB variant, PorB 1A, is correlated with a greater risk of developing disseminated infection. This was shown by Brunham and colleagues; in a study of 325 gonococcal isolates associated with infection, all five cases of DGI were caused by PorB 1A expressing strains, while no DGI cases were caused by PorB 1B expressing strains [7]. This was further confirmed by Illumina sequencing of gonococci from 45 cases of DGI; over 85 % of DGI isolates possessed PorB 1A alleles [35]. This may be associated with complement resistance conferred by PorB; while both variants bind C4BP (if via different mechanisms) [36, 37], PorB 1A is also able to bind factor H, a complement regulatory protein of the alternative pathway present in human plasma with binding increasing redundancy in complement degradation [38].

Finally, other manifestations of infection, such as gonococcal pharyngitis, are likely linked to the route of infection (oro-genital) [5, 39], with reports indicating that DGI could stem from isolated pharyngitis [1, 2, 39]. Another largely overlooked type of infection is ophthalmia neonatorum. It is caused by infection of the newborn during passage through the birth canal of an infected mother, and if left untreated can cause ulceration and perforation of the globe of the eye [40]. Adult gonococcal conjunctivitis caused by contact with sexual secretions is even more under-recognized; while rare, the consequences of untreated infection are the same as in neonates [41]. There is a lack of research into the receptors for adhesion and fitness of gonococci persisting on the eye, which may lead to permanent blindness [1, 2].

Thus, a number of different clinical manifestations can be induced by the gonococcus. However, are all gonococci equally able to cause PID, DGI, neonatal infections, pharyngitis and/or asymptomatic infections? Are there genetic determinants that facilitate the ability of the gonococcus to survive and persist in different anatomical sites – with variation between strains potentially pivotal in improving our understanding of the biology of this bacterium? Evidence suggests that a mix of mechanical routes of entry and genetic determinants impact establishment in different niches.

## Gonococcal virulence factors are diverse, which may influence pathogenesis

### The 'pathogenome' is difficult to define in *Neisseria* spp.

*N. gonorrhoeae* is a primary human pathogen colonizing the genitourinary tract [1]. This is in contrast to other human-associated *Neisseria* spp., which are either commensal bacteria of the nasopharynx (most of the *Neisseria* spp.) or opportunistic pathogens [42]. The best described is *Neisseria meningitidis *, which normally colonizes the nasopharynx of approximately 10 % of the adult population, but may become invasive causing life-threatening septicaemia and meningitis [43–45]. This difference in tropism and life cycle suggests that the gonococcus has diverged significantly from other *Neisseria* spp., which is reflected in bacterial genome content.

To understand the evolutionary history of *Neisseria* spp., it is vital to examine their genomic organization. *N. gonorrhoeae* is interesting in that it is polyploid, with three recognized plasmids (of which one is conjugative and one mobilizable), often in large copy numbers, which facilitate horizontal gene transfer [46]. The rate of genetic variation and genome reorganization is increased further by natural transformation. This capacity is enhanced for DNA originating from other gonococci distinguished by the DNA uptake sequence 5′- ATGCCGTCTGAA-3′ (underlined bases are semi-conserved between strains) [47]. To investigate lineages and evolution, several methods have been developed. Initially, meningococcal diversity was described using multilocus sequence typing (MLST), which uses seven housekeeping gene sequences to group strains into ‘clonal complexes’ often of similar biology. However, for gonococci with much higher horizontal gene transfer rates [48], MLST is insufficient to distinguish strains; strains from the same sequence type (ST) often have significantly different genome ancestry. The first directed attempt was NG-MAST, which enables identification of transmission clusters using polymorphisms in TbpB and PorB [49]. Currently, the most common method is core-genome MLST (cgMLST), which utilizes over 1600 core-genome genes to subgroup gonococcal strains into STs and lineages [50]. The division of gonococci into lineages with semi-clonal structure allows the approximation of gonococcal population dynamics [51].

Genetic variation of multiple genes in *Neisseria* spp. is increased by phase variation. It can be defined as a phenomenon in which genes undergo a reversible switch in their expression state. Most commonly, it is mediated by a homopolymeric tract consisting of simple repetitive sequences, such as a poly-G tract or short tandem repeat, either in the open reading frame (ORF) or the promoter region [52]. Due to the repetitiveness of the sequence, during DNA replication, the polymerase is prone to slipped-strand mispairing, resulting in either an increase or decrease in length of the track amplified. The outcome depends on the location of the homopolymeric tract. In the promoter region, an increase in the distance between promoter and ORF usually modulates the expression level of the protein; in contrast, phase variation within an ORF usually results in switching expression ON or OFF as it is brought either in or out of frame [52–54]. Alternative mechanisms of phase variation are invertible DNA sequences or altered DNA methylation states; in *Neisseria* spp., these are minor mechanisms and the phase variation of factors discussed in this review is mediated by slipped-strand mispairing [52].

Studies investigating the genetic lineage structure and evolution of the genus *Neisseria* have indicated that the two pathogenic species, *N. gonorrhoeae* and *N. meningitidis *, although evolutionarily separated, likely diverged from a common ancestor. This was followed by a change of tropism of the gonococcus to a different anatomical site, which has further driven the separation of the two species. Bennett *et al.* used whole-genome analysis to show that approximtely 60 % of the core-genome content is shared between commensal *Neisseria lactamica* and pathogenic *N. meningitidis* and *N. gonorrhoeae *, with distinct patterns of genetic variation specific to individual species suggesting little evidence of recombination and evolutionary separation [48]. Similarly, MLST approaches, either based on 51 ribosomal loci (rMLST) or 1114 core-genome loci (cgMLST) phylogeny analysis, highlight a clear separation of pathogenic *Neisseria* from commensal species, followed by a subsequent evolutionary separation of *N. gonorrhoeae* and *N. meningitidis *; this can be contrasted with *Neisseria polysaccharea* or '*Neisseria bergeri'*, which consist of distinct lineages, leading to suggestion of separating *N. polysaccharea* into separate species [55]. This means that the majority of the core genome is shared across the genus *Neisseria *, making the identification of genetic determinants implicitly associated with a particular disease phenotype challenging. For example, several studies have shown that many virulence determinants are shared between both pathogenic *Neisseria* species and commensals; e.g. one can find the gonococcal genetic island in *N. meningitidis *, capsule biosynthesis (*cps*) loci in some commensal *Neisseria *; similarly, iron transport systems like FetB or TbpB or adhesion structures such as type IV pili are present in both pathogenic *Neisseria* and commensals [56–59].

One exception to that rule is the recently identified meningococcal clade US_NmUC. It has a particular propensity for causing urethritis, and interestingly has acquired a unique, recombined, non-functional factor H binding protein (which in meningococci serves to protect against complement deposition by recruitment of factor H, which in turn inhibits the alternative complement pathway) [60]. It has also lost the capacity for capsule synthesis due to multi-gene deletion in the *cps* locus and acquired several gonococcal alleles including the anaerobic respiration cluster *aniA*/*norB* [61–64]. Firstly, this indicates that recombination of the core genome between meningococci and gonococci is possible once the anatomical barrier is removed. Interestingly, such genetic transfer events are yet to be identified in gonococcal pharyngitis, where the anatomical barrier is also absent. Second, due to its recent emergence, this clade may highlight which genes are necessary for pathogenesis at this anatomical site and should be closely monitored. Still, it is apparent that examination of the overall genome content is not sufficient to understand the different disease phenotypes exhibited by the gonococcus and meningococcus, and that alternative approaches are needed to define the gonococcal pathogenome and how it differs from commensal *Neisseria* and *N. meningitidis *.

### Genetic diversity of virulence factors has been unevenly investigated

**Fig. 1.**
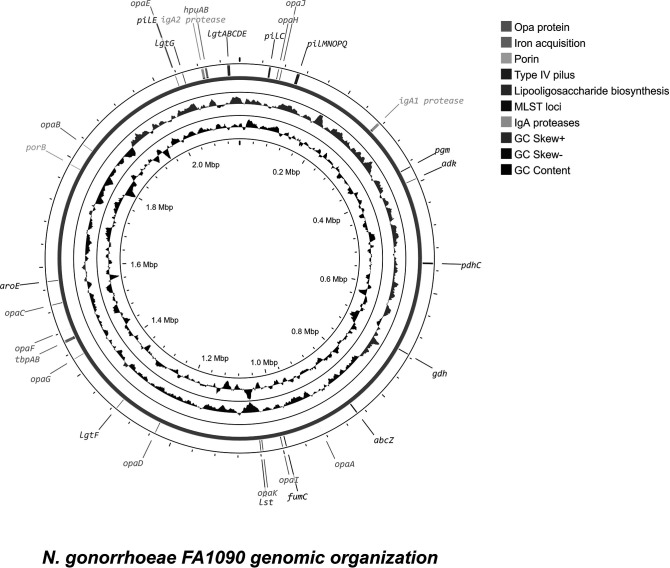
Visualization of the bacterial chromosome of *N. gonorrhoeae* FA1090 strain with a selection of confirmed and putative virulence factors marked. G+C content is indicated with a black wave; positive G+C skew is indicated in green, while negative G+C skew is indicated with purple waves. Key variable genes involved in pathogenesis are annotated with arrows and gene names. Colours indicate a process relevant for pathogenesis in which the gene is implicated as per the key. For reference, MLST loci are annotated with arrows and gene names in purple.

*N. gonorrhoeae* is a mucosal pathogen with infection characterized by efficient pathogenesis and a plethora of genetic determinants facilitating these processes (Fig. 1, Table 2). Understanding these determinants – with variation between strains being potentially pivotal for our understanding of gonococcal biology – will provide opportunities to develop new preventative and therapeutic strategies. What is known about the genetic variation of particular virulence determinants implicated in pathogenicity will be discussed here.

**Table 2.** Nomenclature of a selection of major virulence factors in *N. gonorrhoeae
* The designations were accessed from the PubMLST database, which contains a complete list of gonococcal loci [169].

Currently used name	Abbreviation	Alias and historic nomenclature	NEIS designation (gene name)	NGO designation
Porin B	PorB	Protein I	NEIS2020	NGO1812
Lipooligosaccharide	LOS	Lipopolysaccharide (LPS)	NEIS1902 (*lgtA*), NEIS1901 (*lgtB*), NEIS2154 (*lgtC*), NEIS2155 (*lgtD*), NEIS1900 (*lgtE*), NEIS1618 (*lgtF*), NEIS2011 (*lgtG*)	NGO1353 (*lgtF*), rest undesignated
Opacity protein	Opa	Protein II	NEIS1403 (*opaA*), NEIS 1551 (*opaB*) and others	Undesignated
Type IV pili	T4P	–	NEIS0210 (*pilE*), NEIS0408 (*pilQ*), NEIS0371 (*pilC*), silent *pilS* cassettes (unassigned) and others	*pilE* and *pilS* cassettes – undesignated, NGO0094 (*pilQ*), NGO055 (*pilC*) and others
IgA proteases 1 and 2	IgA1, IgA2	–	NEIS1959 (*iga2*), NEIS0651 (IgA1 protease)	NGO2105 (*iga2*), NGO0275 (IgA1 protease)

### Lipooligosaccharide (LOS) and sialylation

*N. gonorrhoeae* is a Gram-negative bacterium and, similarly to most Gram-negative bacteria, requires lipopolysaccharide (LPS) to maintain outer membrane integrity. In most Gram-negative bacteria, LPS consists of three main domains: lipid A, a canonical Toll-like receptor 4 (TLR4) agonist; an oligosaccharide core that is often variable within and between bacterial species; and O-antigen, which is a polysaccharide extending over several nanometres from the bacterial cell surface [65]. *Neisseria* are an interesting exception to this rule – they do not possess the genes required for O-antigen synthesis, rendering their LPS unusually short (‘rough’ in opposition to ‘smooth’ O-antigen containing LPS) [66]. As a result, ‘poly’ is sometimes deemed inappropriate to describe *Neisseria* LPS and LOS is used instead [14, 67].

**Fig. 2.**
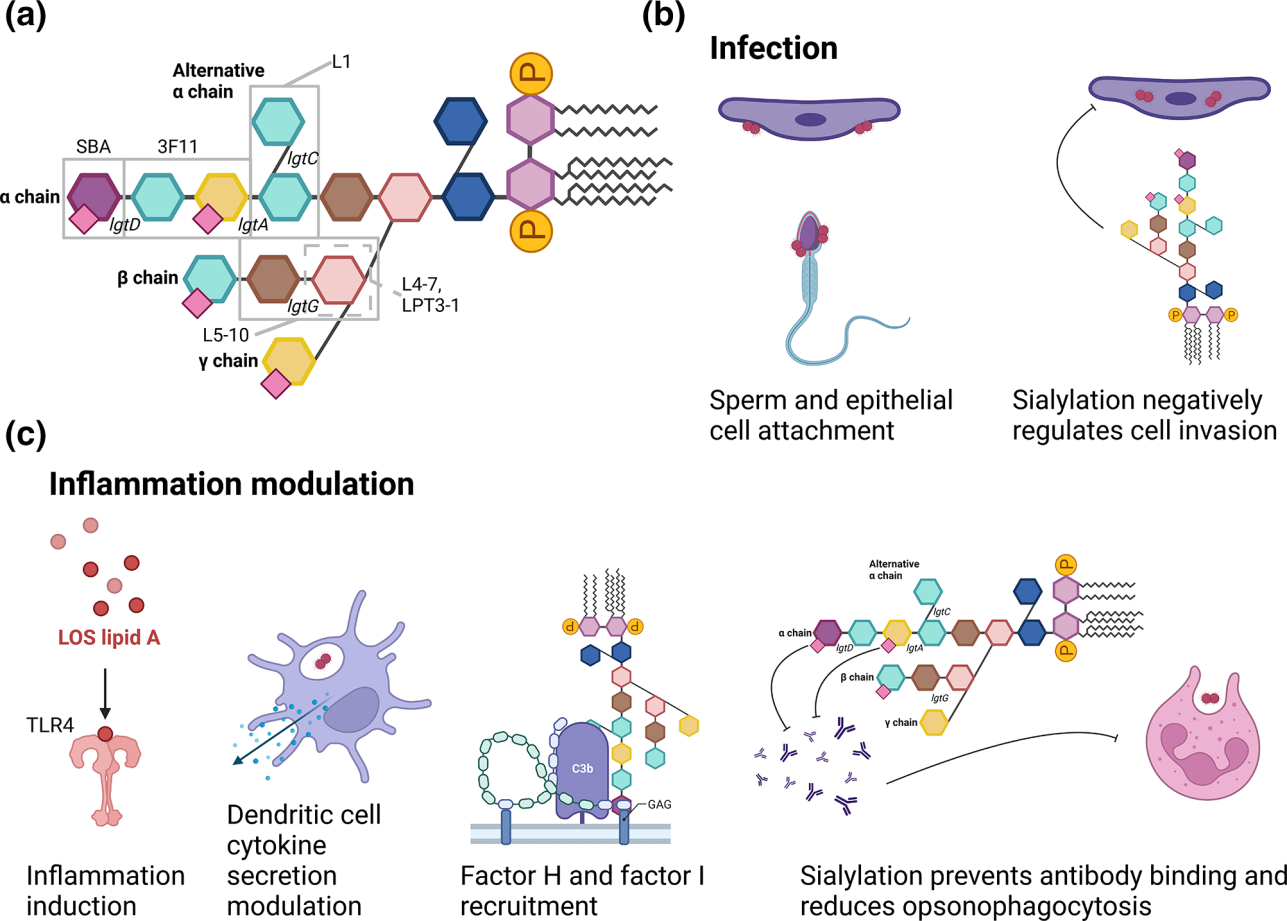
Schematic representation of roles of LOS in infection, invasion and immune evasion of *N. gonorrhoeae *. (a) Schematic representation of fully extended LOS molecule. Oligosaccharide chain names are indicated in bold. The grey rectangles indicate epitopes of labelled monoclonal antibodies against LOS. Names of phase variable LOS biosynthesis genes are indicated in italics next to the bond they synthesize [14, 67, 70, 71, 181]. (b) Roles of LOS in infection. Visualization of LOS-mediated sperm and epithelial cell attachment and negative impact on invasion of host epithelial cells. (c) Roles of LOS in inflammation modulation. Visualization of LOS mediated Toll-like receptor 4 (TLR4) activation, modulation of dendritic cell cytokine secretion, factor H and factor I recruitment and sialylation- and phase-variation-mediated escape from humoral immunity and opsonophagocytosis. Pink squares, sialylation sites. Created with BioRender.com.

LOS biosynthesis genes are mostly conserved across the genus *Neisseria *. The main biosynthetic genes are encoded by the *lgtABCDE* locus, with *lgtF* and *lgtG* located in separate locations on the chromosome (Fig. 1) [54, 68]. Conservation of locus presence across the genus does not imply nucleotide sequence homology between species or indeed between strains of a species, and four *lgt* genes responsible for synthesis of outer core LOS chains are phase variable. In these loci, homopolymeric tracts within the ORF elongate or shorten due to slipped-strand mispairing, resulting in a gene becoming in phase or out of phase and antigenic variation on the gonococcal cell surface (Fig. 2a) [54, 67, 69].

Phase variation of LOS is interesting in terms of virulence. It is known that *N. gonorrhoeae* LOS core is an immunogenic structure, with antibodies developed against individual components of the molecule [70–72]. LOS oligosaccharide is not essential for gonococcal viability *in vitro* [54, 71, 73]. However, antigenic variation, achieved through phase variation, allows the gonococcus to escape the adaptive humoral immune response but can prevent its attachment to sperm cells and male urethral epithelial cells if *lgtA* phase varies OFF due to reduced engagement of asialoglycoprotein (ASGP-R) by the LOS α chain [74, 75]. This would suggest that *lgtA* is important for male urethral infection and transmission. Recently, phase variable *lgtD* has been shown to prevent the addition of terminal KDO residues in unsialylated LOS, which may have an impact on therapeutic approaches [14]. Little is known, however, on the function of *lgtC* and *lgtG* phase variation in pathogenesis. Nevertheless, it has been shown that LOS sialylation on an α-chain lactose-N-neotetraose (*lgtA* dependent) recruits factor H, factor I and sequesters C3b, preventing complement activation, which would be lethal for the invading bacterium [76–82]. LOS can potentially induce actin reorganization in host epithelial cells, allowing for Opa-independent invasion [83]. Thus, blockade of this molecule by neutralizing antibodies could seriously hinder gonococcal virulence.

Another aspect of gonococcal LOS impacting pathology is sialylation, a process in which α2,3 and α2,6 linked sialic acid molecules are added to gonococcal LOS by an outer membrane localized sialyltransferase [84, 85]. Sialylation is thought to induce host mimicry of human epithelial surface glycoproteins (which commonly terminate with α2,3 sialic acid residues), and binding of multiple LOS antibodies (e.g. 3F11) is completely abolished by this immune evasion mechanism [86]. Importantly, sialylation occurs in α2,3 pattern only on the lacto-N-neotetraose moiety that is *lgtA* dependent; other linkages are α2,6 and were not observed to reduce anti-LOS antibody binding [85, 87, 88].

With a recent article suggesting an additional ketodeoxyoctonic (KDO) transferase function for Lst, the incompleteness of our current understanding of LOS structure has been highlighted [14]. The LOS structure is also linked to skewed dendritic cell activation profile and neutrophil opsonophagocytosis susceptibility [78, 89] with sialylation significantly decreasing the propensity for cellular invasion and infection, steps vital in the development of DGI [74, 76, 90–93]. These data are consistent with LOS being an important molecule that is highly variable throughout the course of infection (Fig. 2a, b, c). All of this, combined with the abundance of the molecule in the membrane, makes it an important virulence determinant – which may have activities that are yet to be described.

### Outer membrane protein PorB

*N. gonorrhoeae* possesses several proteins in its outer membrane, with the porin PorB the best described and most abundant [94]. It is encoded by a single gene that has two main variants, PorB 1A and PorB 1B, which differ by which portion of the protein is surface exposed and size (34–36.5 and 36–38 kDa, respectively) [94, 95]. Typically, the gonococcus expresses one variant only, which accounts for approximately 60 % of all outer membrane proteins. It forms a trimeric β-barrel porin, capable of conducting both anions and cations [96]. PorB is widely considered essential for the gonococcus, such that attempts to delete the gene have failed so far. Individual amino acid substitutions are possible in 308 of 328 amino acids of the PorB 1B allele of the FA1090 strain; the non-mutable amino acids were spread across the sequence, with the majority located within the β-barrel and some within extracellular loops [97], indicating that, despite the essential role of PorB in bacterial fitness, it can accommodate extensive non-synonymous genetic changes with unknown consequences for pathogenesis.

Nonetheless, PorB is a major gonococcal virulence factor facilitating immune evasion. Apart from serving as a decoy for antibodies, both PorB variants recruit C4 binding protein (C4BP) [77], which inhibits the classical pathway of complement activation – subverting a significant portion of innate immunity. In addition, the PorB 1A variant is more prevalent in DGI, indicating higher ‘invasiveness’, i.e. propensity to cause severe gonococcal disease compared to PorB 1B (associated with localized infection) [7, 33–35, 98].

One suggested mechanism is significantly better factor H binding to PorB 1A resulting in inactivation of over 75 % of C3b to iC3b, protecting the bacterium from complement activation [80, 88]. Furthermore, PorB can dislocate and insert into host cells; this has been associated with inhibition of neutrophil killing of the gonococcus, exocytosis of endosomes, as well as inhibition of apoptosis of infected host cells [99–104]. Overall, PorB is one of the key virulence determinants in gonococci, providing multiple avenues of immune evasion – yet, the importance of other properties, such as ability to conduce ions, is yet to be fully uncovered.

In terms of clinical practice, the abundance and essential nature of PorB makes it an attractive vaccine target, with several studies showing that monoclonal antibodies binding to PorB prevent infection *in vitro* [105–107]. However, PorB is highly variable [108, 109], with evidence of mosaicism and recombination between strains [110, 111]. This subverts antibody-dependent immunity and is further challenged through complex interactions of PorB with other outer membrane structures such as LOS or RmpM (protein III) [94].

### Outer membrane adhesion protein Opa (protein II)

Opacity (Opa) protein describes the phenotype it induces – Opa^+^ colonies appear opaque on transparent medium [112]. The protein structure is quite variable with conserved, semivariable and two hypervariable regions that aid in immune evasion by antigenic variation. Gonococci possess up to 11 loci encoding Opas, which are subject to extensive phase variation. Since the frameshift events are independent of one another, the bacterium can theoretically express anything from 0 to 11 different Opa variants at any time [113, 114]. The engineering of a strain deficient for all *opa* genes facilitates the study of this family of proteins [115]. Still, most of the research seems to focus on the 11 phase variants of Opa. In addition, the impact of variation of individual Opa loci on pathogenesis is still unknown.

In terms of pathogenesis, Opas serve a very diverse set of functions. One that has been well described is CEACAM (CD66) binding, likely strengthening gonococcal adhesion to host cell surfaces vital for its tropism [116, 117]. Changes in Opa status may correlate with invasiveness in PID as CEACAMs are differentially expressed in the lower and upper female genital tracts [32]. Importantly, due to phase variation of Opa, an infected individual may carry bacteria expressing different Opa profiles, allowing spread of some bacteria while retaining the original infection site. This is consistent with the finding that isolates from the blood of patients with DGI did not express Opa, unlike their counterparts from the urethral or cervical tract, which were able to invade epithelial cell layers *in vitro* [118]. Importantly, four out of five strain pairs isolated from single individuals were not coinfections with two independent strains [118]. Secondly, Opa proteins have been implicated in immune evasion. One component is phase variation, escaping antibody immunity should it develop. However, Opa-treated dendritic cells are unable to activate T cells against their cognate antigen [in this experiment HIV peptides in HIV positive, antiretroviral therapy (ART)-treated patients] [119]. This may contribute to the difficulty of inducing a protective immune response against the gonococcus. Finally, distinct Opa variants confer further properties, such as serum resistance or host-cell killing [117, 120–123]. In general, Opa seems to be exquisitely linked to immune evasion in addition to cell attachment.

### IgA proteases

IgA proteases are secreted proteins that undergo further autoproteolysis until a fully functional protein is produced [124–126]. This protein cleaves mucosal IgA1, which can prevent gonococcal cell attachment and induce opsonophagocytosis [127]. Furthermore, IgA1 protease cleaves LAMP1, disturbing lysosomal homeostasis and, thus, promoting intracellular survival and invasion [128–130].

Genetic variation of IgA protease indicates a mosaic composition mediated by horizontal gene transfer [131, 132]. Evidence regarding the essentiality of IgA1 protease for virulence is uncertain; it has a clearly defined mechanism of action, being important *in vitro* in epithelial cell invasion as well as being strongly associated with pathogenic but not commensal *Neisseria *. However, experimental infections of both female mouse models and human male volunteers show that IgA1 protease mutants can establish infection [133, 134]. It is important to note IgA2 was not investigated in these studies, and a relatively brief infection may have underestimated the importance of antibody cleavage activity. Although less studied, there is evidence to suggest that IgA2 protease undergoes phase variation, suggesting susceptibility to immune detection [59].

### Outer membrane adhesion organelles: type IV pili

The gonococcus possesses characteristic structures known as type IV pili. They form long filamentous structures, recently imaged at sub-nanometre resolution using cryogenic electron microscopy [135], and are involved in cell attachment, twitching mobility, DNA transformation and microcolony formation, making them a major virulence factor [136]. These structures consist of multiple proteins: membrane spanning PilQ at the base, through which the major pilin subunit PilE in helical polymers forms the majority of the pilus. In addition, PilC is the likely tip adhesin (although its precise location is uncertain), while PilV is a fibre-like pilin subunit, both vital for adherence to epithelial cells [137–139].

The major role of the type IV pilus is attachment of the gonococcus to host cells in an Opa-independent manner; this is how Opa-negative cells retain virulence. This attachment is dependent on pilin-associated proteins PilC and PilV [139]. Once attached, type IV pilus undergoes retraction by the PilT motor subunit. This is a reversible process, and pili can be extended or retracted at any given time [140]. In addition to a role in cellular attachment and motility, this is proposed to modulate inflammation of infected cells. Some groups postulate that CD46 (complement regulatory protein that cleaves C3b and C4b) may be detached from the cells by type IV pilus retraction, modulating inflammation; this remains controversial [141]. Furthermore, CD4^+^ T cells have been observed to be activated by type IV pilus, resulting in secretion of anti-inflammatory IL-10 cytokine, and the pilus has been observed to bind C4BP vital for complement evasion [142, 143].

Pilin antigenic variation has been studied relatively well. The *pilE* major expressed subunit is only one of the many variants carried on the chromosome; non-expressed variants collectively called *pilS* are also carried. There are frequently several *pilS* loci; *N. gonorrhoeae* FA1090 contains 19 unique *pilS* genes in six separate chromosomal locations [144]. Variation results when part of or the entire *pilS* is transferred via a RecA-mediated recombination event into the *pilE* expression locus forming a new *pilE* variant. Both *pilE* and *pilS* consist of variable portions flanked by conserved portions; conserved sequences serve as a basis for recombination, which leads to insertion of new *pilS* into the *pilE* locus [145]. These *pilE* variants differ in their ability to be expressed and assembled; if they cannot be assembled into functional pili, this results in a non-piliated gonococcus (P^−^); the rate of variation is one of the highest in any bacterium [146]. In addition to amino acid substitutions, antigenic variation is achieved by differential ability to undergo modification by addition of phosphocholine and/or phosphoethanolamine groups [147]. Unlike phase variable proteins, which depend on slipped-strand mispairing, several DNA modification and repair mechanisms are involved in type IV pili antigenic variation, including RecA, RecQ, RecO, RecR, RecJ (RecF like pathway), RecX regulator, growth regulator RdgC, Rep helicase and Holliday junction processing enzymes RuvB and RecG [146]. The inter-strain variation in *pilE* and *pilS* remains understudied, especially in the context of correlation with pathogenesis and invasiveness; though with such a high intra-strain variability, it is difficult to predict how significant such variation would actually be.

### Identification of new virulence factors – where could we look next?

There is significant scope for research into genetic variation of other, perhaps less prominent virulence factors, such as metabolic genes, which may uncover new pathogenesis mechanisms based on relative variability of proteins and regions within proteins. Genome-wide studies, such as a recent bioinformatic analysis by Bayliss and colleagues, describe the full phase variome (all phase variable genes) in gonococci and other *Neisseria* and may foster such discoveries [148].

The gonococcus possesses efflux pumps on its surface, with the best described the MtrCDE system with its repressor MtrR; *mtrR* mutations induce increased resistance to β-lactams [149, 150]. Another such system is FarAB–MtrE. Initially studied for their role in therapeutic antimicrobial efflux, they were soon found to be able to export human mucosal antimicrobial fatty acids. Both MtrCDE system and FarAB increase the minimum inhibitory concentration (MIC) of antibacterial fatty acids [150]. A study by Jerse and colleagues confirmed that a functional MtrCDE system increases bacterial survival in a female murine reproductive tract; FarAB did not confer competitive advantage *in vivo* [151]. On the contrary, a recent genome-wide association study (GWAS) confirmed enrichment of loss of function mutations in the MtrCDE pump components in cervical compared to urethral isolates, both for *N. gonorrhoeae* and urogenitally adapted *N. meningitidis* [152]. Still, one has to note that the gonococcus is a human-adapted pathogen; it may be that the effect of FarAB is detected only when faced with human-specific antimicrobial fatty acids. Furthermore, the pumps seem to have similar roles, and redundancy cannot be excluded.

In addition to expulsion of innate immune antimicrobials, gonococci are also able to inactivate some of these. For example, the gene *katA* encodes catalase, which deactivates reactive oxygen species and to some extent reactive nitrogen species – two important bacterial killing mechanisms employed by neutrophils and macrophages, respectively [153]. Another similar mechanism is ADP-ribosylation by NarE – a protein that only recently has been described in the gonococcus. Discovered to be a truncated variant of ADP-ribosyltransferase present in meningococci, it is able to ADP ribosylate human defensins HBD1, HBD2 and HBD4 [154]. However, all of the studies have only been performed with purified NarE [154]. Finally, the macrophage infectivity potentiator (Mip) protein was found in at least 20 gonococci by Starnino *et al.* [155]. Interestingly, the function is largely unknown except for the fact that the protein is a peptidyl-prolyl cis/trans isomerase (PPIase) that enhances macrophage but not epithelial cell infection [156].

Another interaction has been described that evades simplistic infection paradigms. Typically, pathogens subvert immune activation for their success. However, *N. gonorrhoeae* is associated with significant pyogenic inflammation (neutrophilic influx). Consistent with this, the gonococcus expresses lytic transglycosylases LtgA and LtgD. These enzymes break down cell wall peptidoglycan. Peptidoglycan fragments can act as cytotoxic NOD1/2 agonists, triggering inflammation, resulting in increased neutrophil influx to further disease-promoting inflammation [157]. If intentional, it suggests that immune evasion is not always in the best interest of the bacterium; some suggest that inflammation helps gonococcal spread. Even more interestingly, gonococcal LtgA and LtgD can aid survival in human neutrophils, indicating that perhaps immune overstimulation may be inhibitory [158].

Gonococcal metabolism may also have important implications in pathogenesis. Initial research begun in the second half of 20th century with the definition of gonococcal auxotypes. The nutrients used for auxotyping included ornithine, arginine, proline, uracil, hypoxanthine, leucine and methionine. Several auxotypes were defined; however, little association was found between different auxotypes and gonococcal pathogenesis [7, 159–161]. One exception to this is a correlation between DGI and the arginine, hypoxanthine, uracil (AHU)^−^ auxotype with unknown causal mechanisms [162, 163]; another is dependence on lactate metabolism for long-term survival in human cervical epithelial cells, potentially vital for invasion and spread [164]. This is biologically plausible as an important metabolic pathway, especially that lactate is readily available in the female genital tract from commensal lactobacilli [164].

One metabolism-related mechanism vital for bacterial success is iron uptake. While the gonococcus lacks siderophores, it can obtain iron from host transferrin, haemoglobin and lactoferrin [165]. In an exemplar fashion, variability of iron-acquisition genes was analysed bioinformatically to reveal diversity of the HpuAB system in addition to previously described HmbR. In contrast to HmbR being present only in pathogenic *Neisseria* with gonococci possessing a non-functional *hmbR* gene, HpuAB is present in a wide range of *Neisseria* species with evidence of significant recombination and immune selection [166]. In particular, HpuA is known to be hypervariable as a consequence of phase variation [167]. TbpB, transferrin capturing protein, is also hypervariable. In contrast, TbpA has been reclassified into three major families, with several variable regions and 638 discovered alleles [59]. These data show that metabolic pathways hide previously under-appreciated insights into gonococcal genomics and pathogenesis – which could make them useful therapeutic and vaccine candidates in the future.

## Concluding remarks – what are the challenges faced by gonococcal research and where should we proceed next?

*N. gonorrhoeae* is a pathogen that escapes many stereotypes. Because of that, development of vaccines and targeted therapeutics has stalled for decades. Virulence factors are being described in increasing detail, both the classical ones like LOS, PorB, Opa or type IV pili, as well as the non-canonical ones like iron-acquisition systems, efflux pumps or other immune modulation mechanisms.

Research into virulence factors has been improved due to the development of new research methods. Since the early 2000s, the development of whole-genome sequencing, including next-generation sequencing, has allowed rapid sequencing of whole bacterial genomes, which demonstrated significant sequence variation between bacterial strains. Several are currently available, and draft genome sequences are available for thousands of isolates in ever-expanding genomic databases such as PubMLST [168, 169]. The recent introduction of a non-polymerase-based method of Nanopore sequencing could potentially resolve difficult regions such as phase variable genes, an approach recently confirmed for yeast oligonucleotide tract repeats [170]. Affordable, reliable sequencing provides plenty of data that can be analysed to devise evolutionary patterns (e.g. as in US_NmUC meningococcal urethritis strain) and associations of particular genes or their polymorphisms with particular disease states. Similarly, whole-genome sequencing is the cornerstone of transcriptomics; the transcriptome of gonococci during mucosal infection was published recently, and similar studies may become very relevant to investigating pathogenesis in the future [171].

Furthermore, experimental approaches are needed. Here, two recent developments are key. One is the establishment of a murine female genital tract model of infection [172]. Prior to that, gonococcal studies could only be done either *in vitro*, from clinical patients and in male volunteers – which made them inherently limited in scope either due to the simplicity of *in vitro* systems or due to ethical concerns associated with human infection. The murine genital tract model has tested several putative virulence factors, novel therapeutics and vaccine candidates [173–175]. It is still limited by strict adaptation of several gonococcal virulence factors to human proteins; this was partially addressed by human transgenic mice, such as the CEACAM transgenic mice mentioned earlier [32, 176]. Another approach to this problem is the use of murine vaginal mucosa explants; they offer the benefit of investigating bacteria in association with complex 3D tissue of epithelial and innate immune cells, while being easier to manipulate than *in vivo* models [177, 178]. On the bacterial side, genetic studies including whole-genome sequencing have pioneered genetic modifications of the bacterium for causal and mechanistic studies into pathogenesis. Of note, Seifert and colleagues are currently developing a library of *N. gonorrhoeae* FA1090 deletion strains for all non-essential genes; once completed, it likely will dramatically increase the pace of research into pathogenesis [179].

However, there are some obstacles that still limit the field. The most important one is the complexity of many of the genes that are studied. Automatic genome annotation on the one hand allows huge amounts of genetic data obtained from isolate whole-genome sequencing to be processed, which is the case for the PubMLST database; at the same time, more difficult templates such as phase variable genes are often not annotated, as it is difficult to devise algorithms that work when genes have premature stop codons. Furthermore, similarity between some genes confuses annotation algorithms, and there are examples of wrongly annotated genes in the published databases. Finally, some of the templates are so complex that short-read sequencing methods such as Illumina sequencing are unable to reliably sequence them; as an example, ends of contigs commonly lie in longer poly-G tracts of phase variable genes. Combined, these make bioinformatic analyses challenging.

A second major issue is limitation in the selection of isolates that are available. Most isolates are cervical (female), urethral (male), anal or sometimes pharyngeal (both). The issue comes with acquiring DGI and PID case strains. For both of these disease manifestations, acquisition of bacterial samples from the site of infection is difficult. In the case of PID, it is very invasive to take samples from the upper genitourinary tract and almost never done. For DGI, the lesions are sometimes cultured; however, it is not uncommon to be unable to isolate viable bacteria directly from infected sites despite other methods, such as nucleic acid amplification tests (NAATs), confirming their presence [3]. Finally, ophthalmia neonatorum seems to have the least isolates classified. To show the scale of the problem, at the beginning of September 2022, out of 18 403 *N. gonorrhoeae* isolates published in the PubMLST database, only 17 had eye listed as their source, and only 5 of these were stated to have been isolated from children below the age of 1 – all coming from a single study based in Lisbon, Portugal [180].

Virulence factor genetic variability is an important area of study that could result in better diagnostics, therapeutics and vaccine development boost. This is more important in this bacterium, which is becoming increasingly dangerous to our communities and disproportionately affects low- and middle-income countries. Therefore, we hope that continued research efforts will help to unravel novel relationships of clinical potential in the future.
